# Integrating training in evidence-based medicine and shared decision-making: a qualitative study of junior doctors and consultants

**DOI:** 10.1186/s12909-024-05409-y

**Published:** 2024-04-18

**Authors:** Mary Simons, Georgia Fisher, Samantha Spanos, Yvonne Zurynski, Andrew Davidson, Marcus Stoodley, Frances Rapport, Louise A. Ellis

**Affiliations:** 1 Centre for Healthcare Resilience and Implementation Science, Australian Institute of Health Innovation, Macquarie University, Sydney, NSW 2109 Australia; 2 Department of Neurosurgery, The Royal Melbourne Hospital, Parkville, VIC 3050 Australia; 3 Department of Clinical Medicine, Macquarie University, Sydney, NSW 2109 Australia; 4 Australian Institute of Health Innovation, 75 Talavera Rd, Macquarie Park, NSW 2109 Australia

**Keywords:** Evidence-based medicine, Shared decision-making, Person-centered care, Medical practice, Medical education

## Abstract

**Background:**

In the past, evidence-based medicine (EBM) and shared decision-making (SDM) have been taught separately in health sciences and medical education. However, recognition is increasing of the importance of EBM training that includes SDM, whereby practitioners incorporate all steps of EBM, including person-centered decision-making using SDM. However, there are few empirical investigations into the benefits of training that integrates EBM and SDM (EBM-SDM) for junior doctors, and their influencing factors. This study aimed to explore how integrated EBM-SDM training can influence junior doctors’ attitudes to and practice of EBM and SDM; to identify the barriers and facilitators associated with junior doctors’ EBM-SDM learning and practice; and to examine how supervising consultants’ attitudes and authority impact on junior doctors’ opportunities for EBM-SDM learning and practice.

**Methods:**

We developed and ran a series of EBM-SDM courses for junior doctors within a private healthcare setting with protected time for educational activities. Using an emergent qualitative design, we first conducted pre- and post-course semi-structured interviews with 12 junior doctors and thematically analysed the influence of an EBM-SDM course on their attitudes and practice of both EBM and SDM, and the barriers and facilitators to the integrated learning and practice of EBM and SDM. Based on the responses of junior doctors, we then conducted interviews with ten of their supervising consultants and used a second thematic analysis to understand the influence of consultants on junior doctors’ EBM-SDM learning and practice.

**Results:**

Junior doctors appreciated EBM-SDM training that involved patient participation. After the training course, they intended to improve their skills in person-centered decision-making including SDM. However, junior doctors identified medical hierarchy, time factors, and lack of prior training as barriers to the learning and practice of EBM-SDM, whilst the private healthcare setting with protected learning time and supportive consultants were considered facilitators. Consultants had mixed attitudes towards EBM and SDM and varied perceptions of the role of junior doctors in either practice, both of which influenced the practice of junior doctors.

**Conclusions:**

These findings suggested that future medical education and research should include training that integrates EBM and SDM that acknowledges the complex environment in which this training must be put into practice, and considers strategies to overcome barriers to the implementation of EBM-SDM learning in practice.

**Supplementary Information:**

The online version contains supplementary material available at 10.1186/s12909-024-05409-y.

## Background

 The practice of evidence-based medicine (EBM) requires clinicians to incorporate their own expertise, the best research evidence, and patient preferences when making decisions about patient care [1]. Since its introduction, approaches to teaching EBM skills have focused on the use of critical appraisal to determine the highest level of evidence, largely overlooking clinician expertise and patient preferences [2, 3] and disregarding the established central role of person-centered care and shared decision-making (SDM), where clinician and patient make care decisions together [4]. This disparate approach may be connected to the way that EBM has been traditionally taught during medical training, where education about person-centered care and SDM has occurred in a separate educational silo to EBM education [2, 5, 6]. In recent years, a potential solution has been proposed: teaching EBM and SDM together, where evidence is applied using SDM skills [7, 8].

Some educators and practitioners have identified the potential benefit of incorporating the principals of SDM into EBM training, so that education centers on the patient as well as the evidence [9, 10]. However, very few published studies provide empirical data on how this can be successfully done [8, 11]. In an Australian study, researchers ran a single EBM-SDM workshop for medical and allied health student-clinicians [12], where SDM was introduced as part of the students’ compulsory EBM course. In this study, participants who underwent SDM training in addition to reading SDM material scored significantly higher on measures of ability, attitudes, and confidence in incorporating SDM into EBM when compared to participants who read SDM material alone. In a more recent study, researchers from the same institution conducted a half-day EBM-SDM workshop to train primary care practitioners in using SDM with EBM to improve decision-making for patient care [13]. In this study, pre- and post- workshop observations of doctors’ skills in SDM were assessed via recorded consultations and pre- and post- workshop attitude questionnaires. The results from this pilot found that participants had increased positive attitudes towards SDM and improved SDM skills immediately after the half-day workshop [13], though the focus of this training was limited to general practice-focused clinical scenarios, did not incorporate a study follow-up, and omitted qualitative participant feedback. More recently, a scoping review of 23 studies found that while there has been increasing recognition by educators of the interdependence between EBM and SDM, only a minority of included studies explicitly incorporated EBM and SDM into training content [8].

We previously conducted a series of EBM training courses for junior doctors during which they were taught to apply evidence using SDM skills, namely, an EBM-SDM course. We ran a pilot mixed-methods evaluation, which indicated that while there was a significant increase in positive attitudes towards EBM after the course, there were also several barriers and facilitators that influenced the potential uptake and practice of EBM and SDM [14]. This is unsurprising, given that EBM training for junior doctors is beset by reports of failure to translate new skills and attitudes into clinical practice [9] and SDM is slow to be taken up among doctors in general [15, 16]. The EBM literature has identified that the main reasons given by junior doctors for not practising EBM included: lack of time to learn [17, 18] or practice EBM [19], workplace culture [20], and lack of prior training [20]. Separate SDM literature has identified that barriers to the practice of SDM perceived by doctors, including junior doctors, included time constraints [21], low levels of patient health literacy [22], workplace culture [23], and no opportunities to learn and practice SDM during clinical practice [24]. However, there are few investigations of barriers to the joint practice of EBM and SDM following their integrated training. As such, there is a need for more comprehensive qualitative evaluations of the outcomes of integrated EBM and SDM training, as well as a more in-depth understanding of the barriers and facilitators to their implementation in clinical practice.

Despite positive attitudinal changes towards EBM-SDM after training [13, 14], it is likely that specific barriers prevent the provision of EBM-SDM training and the translation of new skills into clinical practice. It is important to further understand the nature of these barriers so that the impact of EBM and SDM practice can be fully realised. We were interested in examining the private hospital setting, and specific benefits or barriers this setting could introduce. Also of interest was the composition of junior doctor and consultant participant cohorts where most participants were undertaking surgical specialties or training, and its impact on influencing their responses and outcomes following training. In this study, we conducted interviews with junior doctors both before and after EBM-SDM training, and with their supervising consultants to further understand their perceptions and practice of EBM and SDM, and the associated barriers and facilitators.

### Study aims

This study aimed to answer the following research questions:

1. How does an integrated EBM-SDM course influence junior doctors’ attitudes toward, and practice of, EBM and SDM?
2. What are the barriers to junior doctors’ EBM-SDM learning and practice? What are the facilitators?
3. How do supervising consultants’ attitudes and influence impact on junior doctors’ opportunities for EBM-SDM learning and practice?

## Methods

### Design

This study used an emergent qualitative design where data were collected via semi-structured interviews [25]. Social constructivist theory underpinned our study design to enable the exploration of how junior doctors and consultants created their own meanings, attitudes, and understanding about EBM and SDM, and a deeper understanding of their relationships with each other within this context [26]. The study centered around an EBM-SDM course that we conducted at an academic health sciences center. Phase 1 of this study involved conducting and analysing pre- and post-course interviews with junior doctors to understand their perceived barriers and facilitators to learning and practising EBM-SDM [27]. Thematic analysis of the initial interviews with junior doctors raised questions about the role of supervising consultant doctors in EBM-SDM learning and practice, specifically in terms of their support for training and practice opportunities for junior doctors. Thus, Phase 2 of the study used semi-structured interviews with consultants to further understand how their attitudes and influence might impact junior doctors’ opportunities for EBM-SDM learning and practice.

### Study setting

The EBM-SDM training course took place at an integrated academic health sciences center (MQ Health) on an urban university campus, comprising a university-owned private hospital and specialty outpatient clinics [28]. The course was attended by junior doctors who worked at the center. In the Australian setting, junior doctors include new graduates or interns, residents undertaking prevocational training, registrars who are either accredited with a specialty training program or unaccredited, and fellows who have completed specialty training and are seeking sub-specialty training [29]. The EBM-SDM training course consisted of four 90-minute meetings, and covered all steps of the EBM process and the principles of SDM that are incorporated into the fourth EBM step. The course was conducted over an eight-week period to provide trainees with sufficient time in between meetings for reading, reviewing, and preparing material. The course was conducted five times during this study. Adult learning theory was used as a framework for the problem-based, collaborative learning environment where the teachers facilitated rather than directed learners [30]. During the course, junior doctors used their own patient cases to increase the course relevance to their practice and patient care [31]. Additional File 1 contains details of the structure and content of the EBM-SDM training course.

The junior doctors were on a single-term rotation, where they spent one year at the private hospital before returning to rotations in the public hospital system. They worked alongside a variety of other healthcare professionals, including consultants, allied health professionals, researchers, and educators, and were supervised by consultants, specialists from a range of medical and surgical disciplines, who provided individualised mentoring, opportunities for learning and research, and support to enter specialist training programs in Australia. Junior doctors could also take part in educational activities outside of their supervision with consultants, including the EBM-SDM course, to acquire and practice new skills.

### Participant recruitment

Participants were recruited via purposive sampling [32] where doctors from a range of age groups and training backgrounds were approached to obtain a comprehensive sample. In Phase 1, participants were recruited from the university hospital’s training program for junior doctors. Using examples from the literature [33], an estimated number of 12 to 15 interviewees from the available pool of 30 junior doctors was considered appropriate to provide in-depth data, and to cover all the issues that could arise from interviews pre- and post- EBM-SDM training [32]. In a similar process, for Phase 2 we sought a sample of 10 consultants from the available pool of 20 who had current supervisory roles in the training of junior doctors at MQ Health. The junior doctors were approached as they enrolled in the EBM course, while the consultants were identified from a list of junior doctors’ supervisors provided by the faculty learning and teaching administration team and were sent individual emails inviting them to take part in the study.

### Data collection

#### Demographic data

A demographic survey was developed by four authors (MSi, FR, YZ, AD) and emailed to all consenting participants to record their age group, gender, position, country of medical training, period in which training occurred, and prior education in EBM and SDM.

### Interview schedules

Interview questions were developed by the first author (MSi), then reviewed and amended with members of the author team (AD, FR, YZ). In Phase 1, two interview schedules were developed: pre-course and post-course. The pre-course interviews were designed to establish a pre-intervention baseline and explore how junior doctors understood and used both EBM and SDM, and their prior training experiences in each. The post-course interview questions examined changes in knowledge, attitudes, and practice of EBM and SDM and explored junior doctors’ perceptions of combined EBM-SDM training for learning and practice, their intentions to use knowledge gained, the influence of their supervising consultants on EBM and SDM practice, and possible barriers and facilitators to learning and using EBM. In Phase 2, interviews with consultants were designed to understand how they viewed EBM and SDM in their own practice, and their views on whether junior doctors should practice EBM and SDM. Interview questions also explored consultants’ views and experiences of combined EBM and SDM training, in influencing both clinical practice and medical education. See Additional File 2 for all interview schedules.

#### Interview pilot and sessions

In Phase 1, interview questions were designed and piloted with three junior doctors and were subsequently refined into the final interview schedules. In Phase 2, interviews were piloted with one consultant, after which the questions were modified for use with this cohort. Interviews took place in quiet locations with each junior doctor from 2019 until 2022, and with each consultant during 2021; they were conducted face-to-face in 2019, and via Zoom from 2020 due to the COVID-19 pandemic [34]. Author MSi conducted the interviews as 40-minute sessions. All interviewees were given the option to comment on their interview transcripts and study results. One interviewee returned for a second interview to capture additional data. Observational notes were taken by MSi to capture additional contextual factors (such as tone of voice) to assist with thematic analysis.

### Data analysis

In Phase 1 of the study, junior doctors’ transcripts and field notes were thematically analysed [35, 36] to identify, evaluate and report patterns or themes within the data in relation to the three research questions. The first author (MSi) transcribed and familiarised herself with the data. Iterative generation of codes and themes took place with other members of the authorship team (FR, YZ, LAE, GF, SS). Themes were inductively defined as new codes were generated and all themes and sub-themes were named. Transcripts were re-read, and themes reinterpreted until the team decided that data findings had been accurately described. These themes were then used in Phase 2 of the study as a framework to deductively analyse consultants’ interviews. We also included an ‘Other’ category to code any content that did not fit within the framework, and then inductively analysed this content to capture additional sub-themes from the consultant data.

### Research team and reflexivity

MSi, a higher degree research student, developed and delivered the EBM-SDM training course with two other authors (MSt, AD). MSi also developed the interview schedules (with FR, AD, YZ) and conducted the interviews. All participants were informed of MSi’s involvement in the study. MSi has training qualifications in adult education and qualitative research methods, including group and individual interviewing techniques. She analysed the interview data with other authors (FR, YZ). MSi knew all study participants (except two consultants) through her work as a clinical librarian at Macquarie University and discussed with the other authors how her involvement in the study and familiarity with the participants may influence her perceptions and analysis of the interview data. FR, YZ, and SS are health service researchers, with extensive experience in qualitative research. As non-clinicians, they reflected on their experiences and expectations as patients, and as researchers, and how that may influence their interpretation of the interview data. GF and LAE are allied healthcare professionals by background and researchers who drew on their clinical and research skills and perspectives to interpret the interview data. AD and MSt are neurosurgeons with experience in training junior doctors and an interest in medical education and teaching EBM. They knew several study participants through their clinical and research work.

### Ethical approval and study reporting

Ethics approval was obtained in 2019 to interview junior doctors from Macquarie University Human Research Ethics Committee (# 5201927419929), and in 2021 to add interviews with consultants (Ethics no: 52021274125020). The study was reported using COREQ guidelines (See Related Files). 

## Results

### Demographic information of participants

Demographic details of the junior doctors and consultants who participated in interviews are displayed in Table 1. Of the 30 junior doctors who completed the EBM-SDM training, 12 participated in interviews. Of the 12 participating junior doctors, five were fellows, five were registrars, one was a resident, and one was an intern, and thus the junior doctor cohort represented a range of training levels and experience. Half of the junior doctors undertook their medical training in Australia and around two-thirds had some prior EBM instruction, although none had received training in SDM. Five junior doctors completed both pre and post interviews; those who only completed one interview cited time factors and clinical schedules as reasons for non-completion. Most junior doctors who completed the EBM-SDM training course but not the interviews cited time factors as reasons for their non-participation.

**Table 1:** Demographic details of participants

	**Junior doctors**		**Consultants**	
	***Item***	***Number (%)***	***Item***	***Number (%)***
**Gender**	Male	8 (67)	Male	8 (80)
	Female	4 (33)	Female	2 (20)
**Age group**	< 35	6 (50)	< 40	2 (20)
	36-45	6 (50)	41-60	4 (40)
	>46	0 (0)	>60	4 (40)
**Role**	Intern	1 (8)	Consultant	3 (30)
	Resident	1 (8)	Associate Professor	3 (30)
	Registrar	5 (42)	Professor	4 (40)
	Fellow	5 (42)		
**Discipline**	Neurosurgery	8 (67)	Neurosurgery/spine	3 (30)
	Other Surgery	3 (25)	Other surgery	2 (20)
	Medicine	1 (8)	Medicine	5 (50)
**Country of training**	Australia	6 (50)	Australia	6 (60)
	Europe-UK	3 (25)	Europe-UK	4 (40)
	Asia-Africa	3 (25)		
**Previous EBM training**	Yes	8 (67)	Yes	5 (50)
	No	4 (33)	No	5 (50)
**Previous SDM training**	Yes	0 (0)	Yes	0 (0)
	No	12 (100)	No	10 (100)

Ten consultants participated in interviews. Of these 10 consultants, three were Associate Professors and four were Professors. Five consultants had some prior EBM training, and none had any prior SDM training.

### Themes and sub-themes

The study had three key research questions, and four major themes were identified around those questions. The themes, sub-themes, and links to the research questions are summarised in Table 2. In the following results section, junior doctors’ quotes are indicated with “J” and a number; consultants’ quotes are indicated with “C” and a number.

**Table 2:** Summary of key themes, sub-themes, and links to research questions

Theme	Sub-theme	Group	Brief description
1. EBM training, understanding, and practice.^a,c^	a. Understanding and training in EBM	J	Junior doctor’s pre-course training in EBM was limited, and often anchored to research skills and knowledge-gain. Consultants’ understanding of EBM varied from very little to high level.
	b. Actual and intended practice of EBM	J, C	After the course, junior doctors’ intentions to practice EBM increased.
	c. Junior doctors’ perceived training needs in EBM SDM	J	The EBM-SDM course changed the perceptions of junior doctors about their training needs.
	d. Impact of the medical specialty of consultants	C	Consultants saw major differences between surgery and non-surgery specialties when using EBM.
2. Attitudes to EBM.^a,b,c^	a. Attitudes towards the role of evidence in decision-making	J, C	Junior doctors rated evidence highly in decision-making; consultants had mixed views, often preferring to rely on experience or colleagues
	b. Attitudes towards patient involvement in care decisions	J, C	Junior doctors’ attitudes towards patient involvement increased after the course; consultants varied in their attitudes.
	c. Consultant attitudes towards junior doctors’ practice of EBM	C	Consultants had different views on whether junior doctors should practice EBM.
3. Organisational culture and EBM.^b,c^	a. Public vs. Private healthcare	J, C	All doctors saw different opportunities for EBM in public or private healthcare settings.
	b. Medical hierarchy	J, C	Junior doctors were very aware of the medical hierarchy dominated by consultants.
4. Understanding and practice of SDM and its role in EBM.^a,b,c^	c. Understanding and practicing SDM	J, C	Junior doctors had no understanding of SDM before the course; this changed after the course. Most consultants did not engage with SDM.
	a. Effect of medical hierarchy on junior doctors’ practice of SDM	J	Junior doctors saw hierarchy as a significant barrier to practicing EBM and SDM.
	b. Consultant perceptions of junior doctors’ roles in SDM learning & practice	C	Most consultants saw limited roles for junior doctors in practicing SDM

*EBM* Evidence-based medicine, *SDM* Shared decision-making, *J* Junior doctor, *C* Consultant

^a^Research question 1

^b^Research question 2

^c^Research question 3

### Theme 1: EBM training, understanding, and practice

Four sub-themes were identified that related to perceptions and understanding of EBM training and practice: pre-course understanding and learning EBM, application to practice, training needs of junior doctors, and impact of medical speciality.

#### *Understanding and training in* EBM

Prior to the EBM-SDM course, most junior doctors equated EBM to research skills and knowledge-gain, e.g., *“[EBM] …means medicine that has a foundation in scientific studies that have been rigorously peer reviewed and developed through a scientific method…”* (J3). Some junior doctors linked EBM to a statistical outcome or risk measure, using it to give “*the risks of certain procedures … [and] the risks of conservative management versus operative management”* (J4). Of the six junior doctors that trained in Australia, none recalled EBM training within a clinical setting or taught in a way that directly applied to practice. Instead, they reported that EBM training consisted of isolated lectures or projects: *“but other than that, there was no course for EBM. It’s just lectures when I was in med[ical] school”* (J5).

Five consultants indicated a lack of understanding of EBM practice when asked to prioritise its components: “*Literature-based EBM is the most important, anecdotal or doctors’ experiences is the least important, and what was the third one?”* (C7), whilst others were more aware of EBM theory and practice, particularly as it applied to patient care: “*evidence-based medicine in its foundations is meant to tailor it to the particular patient and it is actually quite flexible”* (C1).

#### Actual and intended practice of EBM

Junior doctors’ understanding of the practice of EBM broadened after the EBM-SDM course and was accompanied by increased acknowledgement of patient involvement in their care. One junior doctor described their increased awareness for future practice: “[the course made me wonder] *how can I convey the message to patients and get them to be involved in deciding the management plan?”* (J5). The greatest barrier to practising EBM was lack of time for learning and practice, with all junior doctors mentioning this during their interviews.

#### Training needs of junior doctors

Prior to the EBM-SDM training course, most junior doctors were looking forward to developing skills in searching and critically appraising evidence: “*I’d like a better understanding of what a good quality study is…if something is a RCT or cohort study that I want to be able to say, this is a good RCT or, this is a good cohort study”* (J4). After the EBM-SDM training course, several junior doctors recommended further training to help them maintain and extend their skills. Some suggested EBM training should be provided for longer and include refresher training, and one suggested giving more emphasis to the SDM component “*because this is the practical part of putting it into our daily life, applying it to patients”* (J5).

#### Impact of the medical speciality of consultants

Consultants’ specialisations impacted their practice of EBM. Those practising as physicians, including a neurologist and cardiologist, reported greater access to high-level evidence and guidelines, with one consultant claiming that “*cardiology is very algorithmic in a lot of ways, and that makes that easier…there’s only so many things you can do…. that kind of distils things”* (C6). Consultants from surgical disciplines reported that lower levels of evidence were often drawn upon for decision-making, because “[in surgery] *the evidence, sometimes is not like hard science…many times we base our decisions on grey literature, or on evidence that we acquire over time…or from the experience of our other senior colleagues”* (C9).

### Theme 2: attitudes towards EBM

Three sub-themes were interpreted within the data relating to attitudes towards EBM: attitudes towards the role of evidence in decision-making, attitudes towards patient involvement in care decisions, and attitudes towards junior doctors’ practice of EBM.

#### Attitudes towards the role of evidence in decision-making

Prior to the EBM-SDM training course, most junior doctors’ attitudes toward EBM were focused on the knowledge they could acquire for decision-making, research, and benchmarking their performance, such as “*recommendations that are based on that evidence to inform medical decision-making”* (J3). After the course junior doctors were keen to practice their new EBM skills that had expanded to include finding and using evidence to explain care issues to patients. *“It [explaining evidence] really makes them [patients] feel as though they’re being actively involved in the actual details of their specific case”* (J3).

Consultant participants frequently discussed the pitfalls of using evidence to inform decisions, with one claiming that *“[EBM has] got enormous weaknesses if people think that there’s evidence for everything; that is too simplistic and left brain”* (C2). Furthermore, decisions were reportedly often informed by “*what you’ve been taught by your people training you and your mentors”* (C5). Two consultants explained how they perceived EBM was negatively changing medical practice: “*[EBM] takes away some of the enjoyment out of practicing medicine individually, in the sense that some of the art has been lost”* (T7). Other consultants pointed out advantages of EBM, including provision of high-quality evidence for decision-making that *“gives me the ability to then converse with patients as to why we do things and why it would be most appropriate”* (C1). Two consultants with prior EBM training discussed the conflict with senior colleagues that can often arise when EBM is practised, one stating that “*sometimes this evidence is not strong enough to change the opinion of some [senior] doctors or surgeons”* (C9).

#### Attitudes towards patient involvement in care decisions

Junior doctors expressed mixed attitudes about patient involvement in decisions. Despite post-training beliefs that patient involvement “*will help to establish…better rapport with patients…because they’re more informed and there’s more trust”* (J3), junior doctors also reported the “*need to simplify things for the patient who makes the decision about their life… other than just giving information”* (J8). Six junior doctors did, however, plan for greater patient involvement after they completed the EBM-SDM course: “*I am now more inclined to include evidence-based discussions…in how I approach decisions that we present to patients…. I wouldn’t have really brought it up as a topic [previously]”* (J3).

Consultants also reported mixed attitudes to patient involvement in their care, with one participant stating that “*it’s good that they’re enthusiastic about it but it’s bad that it’s this sort of modern attitude of ‘my opinion’s as good as your opinion’, even if my opinion is based on social media and newspaper reports”* (C4). Six consultants expressed doubts about patients’ ability to grasp complex medical concepts for decision-making, to “*understand something as much as a clinician who’s been doing it for 10, 20, 30 years”* (C8). Three consultants strongly endorsed patient involvement, mostly believing that “*at the end of the day*…*it’s the patient’s body, that they have to be comfortable with the treatment plan”* (C1).

#### Consultant attitudes towards junior doctors’ practice of EBM

Consultants differed in their opinions on whether junior doctors should practice EBM. Five consultants believed there were few roles for junior doctors in evidence-based decision-making, one stating: “*they practice a very protocol driven medicine. And that’s just historical and that’s probably not a bad thing”* (C2). The other five consultants, in contrast, stated that limited decision-making roles should exist for junior doctors: “*doctors at any stage should be able to assess the patient and so they can influence decision-making, based on that*” (C3).

##### Theme 3. Organisational culture and EBM

Two sub-themes were identified pertaining to the influence of organisational culture on practicing EBM: public versus private healthcare, and medical hierarchy.

#### Public vs. private healthcare

Junior doctors and consultants spoke of differences in EBM learning and practice between public and private healthcare settings. Six junior doctors reported that private healthcare settings, such as the academic health sciences center they were based in, facilitated the practice of EBM, because they had protected time for individual study and educational activities. This did not happen during their public hospital rotations, where junior doctors cited high patient numbers and associated workloads that were prioritised. One such junior doctor stated “*Today I’ve just been allocated a study day… I don’t actually think that happens in public hospitals” (*J4*)*.

Four consultants’ views aligned with those of junior doctors about greater protected time available for learning in private settings. Three consultants stated junior doctors had greater opportunities for patient decision-making in the public system, for example, in the emergency department of public hospitals where “*you see people who are coming in [to the emergency department] and often they’ll see the junior doctors before they even see the senior doctor*” (C6).

#### Medical hierarchy

Junior doctors and some consultants discussed the emphasis placed on following the instructions of the most senior consultants. Six junior doctors reported that they were rarely involved in decision-making, but rather, follow the consultant’s lead, regardless of whether the consultant’s decisions were evidence driven. Prior to the EBM-SDM course one junior doctor stated: “*I think in some of my other terms, if I had asked, they [consultants] would just say “this is just part of my experience”* (J2). She maintained this view after the course, recalling one instance when querying a guideline put in place by a consultant: “*I know as a junior sometimes you get a bit of pushback if what you’re recommending is not guideline driven”* (J2).

Two consultants reported that their decision-making capacity was also restricted by their senior colleagues, one consultant claiming that this was *“the consequence of the traditional school and all the experience, based on the decades of “we always did it like that”* (C9). Another consultant spoke of the difficulties faced by those consultants who completed their medical training before EBM was introduced:

*If you look at some of the older clinicians you can be forgiven for thinking that they’re kind of stuck in, frozen in time, right? And that might be a generational thing, but because of this new focus on evidence-based learning and medicine in the nineties, these clinicians didn’t have the benefit of that.* (C3.)

Three junior doctors reported that hierarchies were evident even among themselves, and not just between junior doctors and consultants, such that accredited registrars or fellows often held greater credibility than less experienced residents, interns, and unaccredited registrars. Two consultants stated that they only worked with fellows, not the more junior ranked doctors, whereas other consultants reported greater inclusivity of all junior doctors during decision-making, one stating: “*I am very, very open to accept the data or opinion [of a junior doctor] because it’s based on something which is more updated than what I know, and this is something that happens”* (C9).

### Theme 4: understanding and practice of SDM and its role in EBM

Three sub-themes were identified relating to the understanding and practice of SDM and its role in EBM: Understanding and practicing SDM, the effect of hierarchy on the practice of person-centered care and SDM, and the role of junior doctors in the learning and practice of SDM.

#### Understanding and practicing SDM

Prior to the EBM-SDM course, four junior doctors could not correctly define what SDM meant, and six described SDM as one-way communication of evidence to patients. After the course, they claimed a greater understanding of SDM as part of person-centered care, and that “*you need to have a good basis in EBM, to actually make sure the patient can be even involved in the discussion. So, the patient understands”* (J4). Seven junior doctors believed that SDM and EBM should be taught together, whereas one did not agree: “*I think we don’t need to explicitly incorporate it, that it’s a given”* (J1). Given that the training level of junior doctors was highly varied (i.e., from intern to fellow), there was variability in how they understood and approached SDM. For example, fellows, the most experienced of the junior doctors, described using evidence to provide recommendations to patients rather than eliciting patient preferences whilst referring to evidence. One fellow stated: *“I think most patients are really welcoming if you tell them that people have done it before, the percentage of people who do good, for example, and those that don’t and they’re willing to accept that”* (J10). Consultants conveyed mixed definitions of SDM; some saw it as informed consent, and others saw it as the transfer of information from doctor to patient. All consultants pointed out the difficulties of SDM, with one highlighting that “*it’s really hard to get somebody to the level where they can make some sort of an educated decision”* (C8). One consultant commented on the differences in attitudes towards SDM between older and younger colleagues: “*younger clinicians are less likely to be as paternalistic [than older consultants], they’re more willing to accept that patients have their own thoughts, even if they’re unconventional and unrealistic”* (C3). Surgeons and surgical trainees, comprising 72% of the study cohort, tended to view EBM and SDM as doctor-driven rather than patient-centered. For example, one neurosurgeon emphasised the important sources of evidence used for patient decisions: *“So I always bring to the patient my experience, I bring the MDT [Multidisciplinary Team] meeting decision … and the literature”* (C9). This contrasted with the perspective of non-surgical consultants. For example, a cardiologist highlighted the central role of the patient in the decision-making process: *“I always think of evidence as the hard science and then for the decision-making process, about the application of that hard science to a particular context and … it’s in that paradigm, that the patient’s point of view is used to temper the evidence that you’re presenting”* (C6).

#### Effect of medical hierarchy on junior doctors’ practice of person-centered care and SDM

Six junior doctors reported that, due to their place in the medical hierarchy, they tended not to practice SDM. One participant stated:

*I actually try to hold off on doing that [practising SDM], personally, just because it’s more of a consultant discussion at that stage. When a consultant leaves the room, the patient does actually have more questions, and sometimes I just reiterate what the consultant has already said.* (J4.)

Ten junior doctors planned to increase their communication and person-centered care skills after the EBM-SDM course, for example, using EBM to find evidence that reassures a patient; skills that could be implemented now and expanded later to incorporate SDM.

#### Consultant perceptions of the role of junior doctors in SDM

Four consultants were of the view that junior doctors should not practice SDM due to their junior level. One consultant reported that junior doctors sometimes played a patient advocate role because they “*often have an insight into some of those other levels [of patient care]”* (C2). Another consultant considered providing junior doctors *“the opportunity to be more involved in that [SDM] discussion”* (C7) but cited time constraints as a barrier.

## Discussion

This study explored how integrated EBM and SDM training can impact attitudes, understanding and practice among junior doctors, and whether the attitudes and practice of their supervising consultants can influence those outcomes. Junior doctors demonstrated significant positive attitude changes towards EBM and SDM after the EBM-SDM course. Prior EBM training (during medical training or afterwards) was mostly didactic and focused on knowledge and skill acquisition which is a common finding in other studies that has not equipped junior doctors to practice EBM confidently in clinical settings [37, 38]. Following our EBM-SDM course, not only did junior doctors’ knowledge and skills improve, but they frequently referred to the benefits of including patients in their discussions about care, which indicated that they had expanded their understanding of EBM to incorporate aspects of person-centered care. Their intentions to be more person-centered were frequently based on using evidence to effectively communicate risks and benefits to patients, rather than having SDM conversations with patients where all options were described, and decisions made together. However, there appeared to be a disconnect between the practice of SDM and the *recognition* of its practice. On several occasions, junior doctors facilitated SDM by answering patient questions after the consultant left the room, or by reiterating what the consultant said, but failed to recognise this as part of a SDM conversation with the patient.

Junior doctors also varied in their attitudes and practices of SDM. The more experienced junior doctors, the five fellows, tended to demonstrate a more doctor-centered rather than patient-centered approach to patient care than the less experienced junior doctors (i.e., residents). Junior doctors were at varying levels of their medical training, some of them closer to consultant-level practitioners than others, and may perceive and think about SDM differently depending on their training cohort. Furthermore, several fellows had worked as consultants in their home countries which may have influenced the doctor-centered patterns of decision-making commonly found among consultants. Thus, our study identified that junior doctors attitudes and practices of SDM are likely due to a lack of specific knowledge and understanding of SDM, limited prior training, as well as cultural conventions that may be associated with time and country of training.

Consultants varied greatly in their understanding of EBM and SDM, and their views on whether either should be practised by junior doctors. Senior consultants who completed medical training before the formal introduction of EBM in the 1990s [39] appeared to be unfamiliar with and less accepting of EBM and SDM and expressed a reluctance for junior doctors to engage in either. In contrast, younger consultants who had prior exposure to EBM training and practice tended to appreciate the benefits of EBM for junior doctors and patients. In another study of junior doctors and senior anaesthetists, interviews indicated there was a link between career stage and workplace settings and EBM attitudes [40]. In this study, senior anaesthetists (consultants) were reluctant to make decisions or change practice based on evidence in preference to their own experience and opinion [40]. Junior doctors regarded this as reluctance to change as due to older age, but the consultants saw it as surrendering their professional autonomy [40]. Thus, there may be a tendency among more senior doctors to resist practising EBM in favour of using their own decision-making preferences, that carry a risk of cognitive bias and are potentially suboptimal or obsolete decisions [40–42]. In addition, some studies have shown senior medical staff (consultants) have very little expertise in SDM with patients, thereby failing to become the role models in EBM-SDM that junior doctors need [43]. Senior doctors have also reported difficulty in using technology thus preferring to ask colleagues for advice [44].

In our study, more senior consultants appeared to dominate the medical workforce hierarchy and exclude junior doctors and patients from decision-making. These consultants believed that decision-making should be underpinned by their experience, knowledge, and their communities of practice. Thus, they did not prioritise decision-making linked to EBM and SDM and consequently educational opportunities for junior doctors under their supervision were reduced. These findings support those of other studies concerning the impact of medical hierarchies on junior medical staff, where power is recognised to sit with senior medical staff positioned at the top of the hierarchy, thereby reducing the autonomy of those positioned lower in the hierarchy, such as junior doctors [40, 45]. This has been reported to be particularly evident in surgical specialties, where decision-making is dominated by senior surgeons’ experience rather than evidence [46]. Junior doctors learn to respect hierarchy from medical school, where they do not challenge authority to avoid unwanted impacts on their training and career progression [47–49]. The well-established medical hierarchy emerged as a barrier preventing junior doctors in our study from using evidence-based decision-making skills learned in the EBM-SDM course, particularly if the evidence contradicted strongly held views and practices of senior consultants.

Of note was that the present study was conducted during the COVID-19 pandemic, a difficult and uncertain time for all medical professionals. In the Australian context, junior doctors have reported restrictive workplace cultures and behaviours, including being overlooked and undervalued by senior doctors, which contributed negatively to their psychological well-being during COVID-19 [49]. This had important implications for doctors’ welfare, workforce retention, and safe patient care that needed to be addressed through “positive workplace cultural interventions to engage, validate and empower junior doctors” [50]. In contrast, junior doctors in our study, and in others, have reported that many consultants and senior medical staff were always supportive and approachable role models, not just during the pandemic, and helped to facilitate their trainees’ well-being and progress [47, 51]. The potential contribution of such role models to facilitate and support EBM and SDM learning and practice may help to overcome some of the associated barriers [52].

Combining EBM and SDM training enabled junior doctors to realise there is more to EBM than the level of evidence, which was what most believed before the training. The combined course enabled them to consider how they would communicate the relevant evidence in a two-way conversation with the patient, and thus situated the principles of EBM within the broader context of patient needs and preferences. Several junior doctors had commented that their awareness and practice of improved communication skills with patients had increased after the course, lending support to the effectiveness of the combined course, and the likelihood that the learnings would be utilised in future. These outcomes also imply that EBM-SDM training has the potential to shift power dynamics within the medical hierarchy through expanding the skillset and abilities of junior doctors.

Another facilitator of combined EBM-SDM learning and practice reported in our study was the capacity of private healthcare facilities in Australia to provide protected time for educational activities. This contrasted with public healthcare facilities, where such opportunities are limited [53]. Our study took place within a neurosurgery department where a half-day is set aside each week for learning and teaching meetings, including the EBM-SDM course. The meetings were co-ordinated by consultants, thereby enabling junior doctors to learn and practice new skills with consultants’ support. In a similar way, consultants who recognise the benefits of EBM and SDM could act as unofficial champions, who provide further learning and teaching opportunities for junior doctors, whilst demonstrating and communicating those benefits to their senior colleagues. The idea of champions comes from literature demonstrating that colleagues or supervisors of junior clinicians can be a great source of assistance and support when it comes to learning and practicing skills associated with EBM [8]. Such champions or role models have been recommended as an integral part of EBM teaching because they demonstrate to learners the ‘how-to’ of the application of EBM principles to clinical practice and individual patients [54]. Within our study, this supportive culture, led by a champion or role model, was very beneficial. One of the neurosurgeon consultants took a keen interest in teaching EBM to junior doctors and he led by example, showing them how to use it in daily practice through patient care consultations, and ward rounds and by leading the EBM-SDM teaching during protected education time. The junior doctors responded with increased motivation to practice their EBM-SDM skills during educational meetings. This opportunity provided by a private healthcare facility could be an exemplar of EBM-SDM education in the Australian context that may be adapted by other institutions.

### Future directions

A lack of prior learning and practice of EBM and SDM concepts among this sample of junior doctors echoes previous calls for improved basic and ongoing training in EBM and SDM skills [8, 55]. The recently updated Australian specialist training program [56] has cited the inclusion of EBM and SDM as separate skill sets, with an emphasis on skills and knowledge acquisition. However, there is now a framework providing core competencies that can underpin an EBM curriculum incorporating SDM [57]. This is a promising initiative that could be adapted and used to meet the needs of institutions whilst identifying and managing barriers and facilitators to the learning and practice of EBM and SDM. Additionally, the capacity of consultants with prior EBM training and experience to act as champions of EBM-SDM could be further explored.

Future research opportunities include evaluation of the impacts of integrated EBM-SDM training content and strategies to determine optimal approaches for educators to adopt in both private and public settings. Future research should also focus on the efficacy of strategies to empower junior doctors to become more independent in using their EBM and SDM skills, such as training champions and consultants who want to help their junior doctor trainees develop skills and experience in EBM and SDM [52, 58]. Finally, further investigation is warranted into the significance of undertaking medical training either before or after the introduction of EBM in the 1990s, and how this impacts the medical hierarchy, EBM-SDM training and practice opportunities for junior doctors, and patient care. These investigations could incorporate other qualitative methods such as ethnography to fully capture perceived dynamics and cultural conventions within medical disciplines.

### Strengths & limitations

This study has contributed to our knowledge of combined EBM-SDM training in the Australian context. A strength of the study was its emergent design, where consultant interviews in Phase 2 were added after data were analysed from junior doctor interviews in Phase 1. This approach enabled consultant interview schedules to further elucidate the barriers and facilitators associated with EBM and SDM learning and practice that emerged during Phase 1. The study was also strengthened by including two diverse, but linked participant groups, the junior doctors, and their supervising consultants, thus facilitating the collection and analysis of more than one source of relevant data that addressed the study aims. However, the study is not without its limitations. First, the modest sample size of the study, exacerbated by COVID-19 restrictions and the impact of the pandemic on the medical workforce, reduces the study’s transferability to other cohorts and contexts. Second, junior doctors’ limited understanding of SDM after the course may reflect a limitation of the course. Although SDM was introduced and discussed in the course, little time was provided for deliberate SDM practice and feedback; an issue that can be rectified in future training and research. Third, more males than females participated in the study which may have influenced the pattern of results and is an area for further research.

## Conclusions

Most junior doctors reported positive attitude changes following EBM-SDM training that encompassed plans to increase patient involvement in their care through better communication and evidence-based shared decision-making. However, time constraints and the influence of the medical hierarchy were significant barriers for most junior doctors when learning and practising EBM and SDM. Despite these barriers, supportive consultants and protected educational time facilitated the learning and practice of EBM and SDM within the context of our study. To counter the reported barriers at our institution there are opportunities available for some consultants to become champions who make protected time available for EBM-SDM learning and practice opportunities. These findings may inform future research and training where integrated EBM and SDM learning and practice could be adapted to the unique contextual and cultural influences of each institution.

### Supplementary Information

**Supplementary Material 1.**

**Supplementary Material 2.**

## Data Availability

The datasets used and/or analysed during the current study are available from the corresponding author on reasonable request.

## Abbreviations

EBM: Evidence-based medicine
SDM: Shared decision-making
COREQ: Consolidated criteria for reporting qualitative research
