# Supplementary material for: Integrating training in evidence-based medicine and shared decision-making: a qualitative study of junior doctors and consultants
Source: BMC Med Educ. 2024 Apr 18;24:418. doi: 10.1186/s12909-024-05409-y (PMC11027546; doi:10.1186/s12909-024-05409-y)
Supplement: Supplementary file 1 — Supplementary Material 1. [file 12909_2024_5409_MOESM1_ESM.docx]

| Additional File 1. Overview of EBM-SDM course modules. | Outcomes | Activities | Evaluation |
| --- | --- | --- | --- |
| Module1:  Introduction to EBM, PCC and SDM. History of EBM | - Describe understanding of EBM, and SDM and their relationship to PCC. - List facilitators and barriers to EBM and SDM practice. - Demonstrate competence in understanding the limitations of EBM to guide decision-making. | Pre-class: readings and view video clips. In class:   - - Introduction to course and assessment task (Module 4)   - Small group and large group discussion of key readings and SDM video clips.   - In pairs, then shared with group: challenges and benefits of EBM and SDM   to their practice | - Answer questions in class (and complete online quiz) about the components and uses of EBM and SDM in PCC. - Contribute to discussion on the challenges and benefits of EBM and SDM. |
| Module 2:  Asking questions and searching the literature. Break question up into searchable parts, search evidence summaries and/or databases. | - Ask searchable questions about a patient problem. - Describe differences between foreground and background questions and the resources searched to answer them. - Search a range of databases and pre-appraised sources of evidence using appropriate search strategies. | Pre-class: Reading: asking clinical questions. Video clips on literature searching.  In class:   - - Demonstration of database searching: Ovid Medline, Embase,   - Demonstration of using pre-appraised evidence: (UpToDate; DynaMed; Therapeutic Guidelines)   - Discussion of assessment task for course: presenting a patient case   Follow up activity: Each participant schedules meeting with librarian to conduct database searches | - Each participant completes and submits a clinical question template on the clinical case chosen to present in module 4 - Participate in database searching tutorial with librarian. |
| Module 3:  Critical appraisal I (Randomized controlled trial) and Critical appraisal II (systematic review and meta-analysis). | - Demonstrate critical appraisal skills in understanding and discussing the quality of published studies. - Apply evidence to a patient care decision using SDM. | Pre-class: Read two papers.  In class:   - - In small groups, use EBM checklists to evaluate a clinical trial and a systematic review.   - Role play: Use results to engage in SDM conversation with a patient. | - Participation in SDM exercise (formative feedback) |
| Module 4:  Assessment task: Present patient case inclusive of all steps of EBM, including SDM. Complete (online) a reflection on the role of EBM and SDM to their practice. | - Demonstrate knowledge and skill in conducting each step of the EBM process - Engage patients in developing plans that reflect their health care needs and goals using SDM. - Document reflections on the ways in which EBM, PCC and SDM skills and knowledge can change clinical practice and patient experiences. | In class:   - Case study presentation using all steps of EBM and description of how clinician would engage in SDM with a patient to reach a healthcare decision. - Peer feedback on presentations. - Group discussion on the role of EBM-SDM training and practice for doctors and patients. | - Summative assessment: class presentation of a patient case, Submission of presentation slides. - Online written reflection on the value of SDM and EBM to doctors’ practice and patient experiences and outcomes. |

EBM: Evidence-based medicine, PCC: Person-centred care, SDM: Shared decision-making
