# Supplementary material for: Integrating training in evidence-based medicine and shared decision-making: a qualitative study of junior doctors and consultants
Source: BMC Med Educ. 2024 Apr 18;24:418. doi: 10.1186/s12909-024-05409-y (PMC11027546; doi:10.1186/s12909-024-05409-y)
Supplement: Supplementary file 2 — Supplementary Material 2. [file 12909_2024_5409_MOESM2_ESM.docx]

Additional File 2 Interview schedules

**INTERVIEW SCHEDULES FOR JUNIOR DOCTORS AND CONSULTANTS**

**1.** Interview Schedule For Junior Doctors

Junior doctors have already signed Participant Information and Consent Forms for Doctors (PICF) agreeing to the interviews.

**Interview format:** Interviews will take place face to face. Each interview will take approximately 40 minutes.

**1a.** Pre-EBM-SDM Course Script For Junior Doctors

Thank you for agreeing to be interviewed today. The purpose of this interview is for us to understand your experiences in using principles of evidence-based medicine and shared decision-making when caring for patients in Macquarie University Hospital. Evidence-Based Medicine (EBM) incorporates best research evidence, the doctor’s experience, and patient preferences when making decisions about patient care.^3^ This interview will be recorded and transcribed into text and your name will be permanently removed to completely de-identify the interview transcript. Only the investigators of the study will have access to the interview transcripts. The interview will take approximately 40 minutes. My details are on the consent form if you wish to contact me at any time about the interview.

Do you have any questions about the study or the interview?

## Pre-Course Questions For Junior Doctors

- How long have you been at MQ Neurosurgery? What stage are you at with your neurosurgery training? (Modify for other disciplines)
- What do you understand by the phrase “the practice of evidence-based medicine?” Can you give me an example of EBM?
- What is your experience of EBM training? [How helpful was it?] [Ask next question if training was undertaken]
- How confident do you feel about using the training you received? [Depending on what they answer, examine why they do or do not feel confident]. Can you give me an example?
- How do you use EBM in your daily routine? Ask for examples.
- How do you think EBM is perceived by your peers and by your supervisors? Why is that?
- What do you understand by the term “Shared Decision-Making”?
- How important do you think SDM is to doctors and their patients?
- Have you received training in SDM? Can you tell me more about that?
- (if they haven’t received SDM training) Do you think it is important for doctors to receive SDM training? Is it something you would be interested in learning more about?
- What do you hope to achieve by doing the EBM workshop? (How will this further your career or support your practice?)
- What, in your experience, are the benefits of EBM? What are the drawbacks? Can you give an example of a situation where you used EBM to good effect, and one where it hindered your work, either in terms of communicating with a patient or managing patient care?

**Wrap Up:** Thank you for participating in this interview. Do you have any questions?

**1b.** Post-EBM-SDM Course Script And Questions For Junior Doctors

## Introduction

Thank you for agreeing to be interviewed today. The purpose of this interview is to establish if the EBM course you completed has helped to change your attitudes towards, and practice of, EBM and SDM with your patients. This interview will be recorded and transcribed into text and your name will be permanently removed to completely de-identify the interview transcript. Only the investigators of the study will have access to the interview transcripts. The interview will take 40 minutes. My details are on the consent form if you wish to contact me at any time about the interview.

Do you have any questions about the study or the interview?

## Questions:

We know that, according to Dave Sackett, Evidence-Based Medicine (EBM) incorporates best research evidence, the doctor’s experience, and patient preferences when making decisions about patient care ^3^

- How do you feel about EBM now that you have finished the program?

(Has the EBM program changed your knowledge of and attitude towards EBM? If so, how?)

- Has the EBM program changed the way you practice medicine? In what ways? Can you give an example of a practice change?
- Has EBM training changed the way you interact with patients? How?

(Have you had an opportunity to apply your EBM knowledge and skills with patients? Can you give me an example?)

- How useful was the SDM section of the course?
- Do you think EBM and SDM training belong together? Why? Why not?
- Have you had an opportunity to hold shared decision-making conversations with your patient? Can you give me an example?
- How did you feel about using a shared decision-making approach in conversation? How do you think the patient felt?
- Do you think EBM and SDM can influence patient care and outcomes? If so, how?
- How would you feel about asking a senior doctor to change their practice on the basis of a new clinical guideline? How would you approach this?
- What problems or barriers have you encountered (or expect to encounter) when practising EBM? Can you suggest ways of overcoming them? (Suggested prompts if needed: For example, lack of time, supervisors who do not practice EBM, misunderstandings of what EBM is, how it can be applied etc.)
- What problems might you encounter using SDM? How could you overcome them? (Patient doesn’t understand risk and benefit, patient doesn’t want to make decision; Using decision aids or other visual prompts to aid understanding);

**Wrap up:** Thank you for your time. Are there any questions you would like to ask?

**2.** Interview Schedule For Consultants

Each senior doctor has signed a Participant Information and Consent Form for Consultants (PICF), agreeing to participate in the study by being interviewed. Interviews will be held within one month following the junior doctors’ completion of the EBM program.

## Script And Questions.

Thank you for agreeing to be interviewed today. The purpose of this interview is for us to understand your views on practising EBM when caring for patients in Macquarie University Hospital. We are also interested in your views on the degree to which junior doctors should practise EBM, including shared decision-making, with the patients under your care and on the values of EBM training for junior doctors. This interview will be recorded and transcribed, and your name will be permanently removed to completely de-identify the interview transcript. Only the investigators of the study will have access to the interview transcripts. The interview will take approximately 40 minutes. My details are on the consent form if you wish to contact me at any time about the interview.

Do you have any questions about the study or the interview?

## Interview Questions For Consultants

- Evidence-Based Medicine (EBM) incorporates best research evidence, the doctor’s experience, and patient preferences when making decisions about patient care.^1^ How much importance do you give to EBM to inform each of these components (best research evidence, the doctor’s skills and experience and patient preferences and values) in your practice?
- To what degree do you think you practice EBM? Can you give an example to illustrate your answer?
- How do you view shared decision-making? Do you see as a complementary part of EBM? Why? Why not?
- What do you think is your role in shared decision-making with the patient? And what is your role in shared decision-making with the junior doctor?
- What do you think the junior doctor’s role is in the decision-making process?
- If a junior doctor asked you to change your practice based on a newly published guideline, how would you respond? Why do you think you would respond in that way?
- What do you think are the strengths and drawbacks of EBM? Can you give an example of a situation that exemplified the strengths and another that exemplified the weaknesses?
- What do you think are the strengths and drawbacks of SDM?
- Do you think SDM and EBM should be seen and taught as complementary parts of the patient-centred care process? Why? Why not?

## Closing Instructions

Thank you for your time. Are there any questions you would like to ask?
